# Systemic Oxy-Inflammatory, Hormonal, and Mood Responses to a Ski Mountaineering Competition: Preliminary Evidence from Military Athletes

**DOI:** 10.3390/sports14090388

**Published:** 2026-09-02

**Authors:** Simona Mrakic-Sposta, Cristina Ranuncoli, Vittore Verratti, Maristella Gussoni, Federica Mrakic-Sposta, Consuelo Rubina Nava, Manuel Marzola, Michela Montorsi, Alessio Cavicchioli, Alessandra Vezzoli

**Affiliations:** 1 Institute of Clinical Physiology, National Research Council (IFC-CNR), 20162 Milan, Italy; maristella.gussoni@unimi.it (M.G.); michela.montorsi@uniroma5.it (M.M.); alessandra.vezzoli@cnr.it (A.V.); 2 Scuola Regionale dello Sport CONI Valle d’Aosta, 11100 Aosta, Italy; 3 Department of Science, University “G. d’Annunzio” Chieti—Pescara, 66100 Chieti, Italy; vittore.verratti@unich.it (V.V.); manuel.marzola@phd.unich.it (M.M.); 4 IRCCS Humanitas Research Hospital, Via Manzoni 56, Rozzano, 20089 Milan, Italy; federica.mrakic_sposta@humanitas.it; 5 Department of Economic and Political Sciences, University Valle d’Aosta, 11100 Aosta, Italy; c.nava@univda.it; 6 Centro Addestramento Alpino dell’Esercito Italiano, 11100 Aosta, Italy; al.cavi@yahoo.it

**Keywords:** oxidative stress, inflammation, endurance exercise, hypoxia, high altitude, mountain, ski mountaineering, military athletes, Trofeo Mezzalama

## Abstract

The study investigated the systemic responses concerning oxy-inflammation, hormones, and mood after a ski mountaineering competition. Nine experienced military ski mountaineers (age, 32.37 ± 8.90 years; body mass, 59.37 ± 9.58 kg; BMI, 19.85 ± 1.53 kg/m^−2^) were non-invasively assessed using saliva and urine samples collected at baseline (T0) and immediately after competition (T1). Reactive Oxygen Species (ROS), total antioxidant capacity, lipid peroxidation (8-isoPGF2α), systemic inflammatory responses (IL-6; TNF-α; sICAM), neurobiological markers of stress and mood regulation (cortisol and serotonin), and indicators of metabolic and immune activation (creatinine, neopterin, and uric acid) were obtained. In addition, psychometric, physical, and subjective rating scales were administered. At T1, significant increases in oxy-inflammatory biomarkers were recorded (ROS: +137%; 8-iso: +121%; IL-6: +175%; TNF-α: +45%); stress hormones (cortisol: +241%; serotonin: +141%), associated with changes in urinary creatinine (+60%) and an increase in immune activation (neopterin: +94%). An exploratory analysis suggested a potential quadratic association between race completion time and post-race ROS production rate (R^2^ = 0.552), warranting further investigation in larger cohorts. At T1, Visual Analog Scale (VAS) scores increased by 98% for tiredness, 112% for fatigue, 36% for happiness, and 320% for pain. In conclusion, ski mountaineering competitions place an extremely high physiological burden on athletes, eliciting complex oxidative, inflammatory, metabolic, hormonal, and psychophysiological responses that may reflect the interactions among exercise intensity, cumulative hypoxic exposure, and redox adaptive mechanisms.

## 1. Introduction

Military athletes and, in general, ski mountaineers are exposed to considerable physiological stress due to prolonged endurance exercise, high-altitude exposure, and rapid changes in environmental temperature [1]. These conditions increase metabolic demand and oxygen consumption, thereby promoting the generation of reactive oxygen species (ROS), which can contribute to oxidative stress and activation of inflammatory pathways [2]. In winter endurance sports, prolonged or repeated high-intensity exercise has been shown to alter antioxidant capacity and oxidative stress biomarkers [3,4]. Studies on elite alpine ski racers during intensive training periods have reported changes in antioxidant status and lipid and protein oxidation markers, suggesting that sustained training loads may challenge the athletes’ antioxidant defense system [4,5,6]. Similarly, ski mountaineering competition, which combines prolonged endurance effort and altitude exposure, has been associated with muscle cell damage, oxidative stress, and hormonal alterations. Increases in markers such as creatine kinase and C-reactive protein, together with elevated cortisol levels, have been observed after multi-day ski mountaineering races, reflecting the physiological strain imposed by this activity [7].

Moreover, in recent years, the scientific literature has increasingly recognized the importance of military sport contexts in relation to performance psychology. Consequently, psychological skills such as resilience, emotional regulation, and stress management have been identified as key determinants for maintaining optimal performance and operational readiness [8,9].

In parallel, increasing attention has been directed toward the role of mental health and well-being in military sport settings. Psychological well-being is now considered a crucial component not only for sustaining athletic performance but also for supporting recovery and long-term health in highly demanding environments [10].

A ski mountaineering competition provides a complex model combining at least four concurrent stressors: prolonged endurance exercise, hypoxic exposure (above 4000 m), abrupt changes in environmental conditions, and competition-related psychological stress [11,12]. High-altitude exposure represents an additional source of oxidative and inflammatory stress beyond the exercise itself [13,14]. In fact, independently of the exercise workload, hypobaric hypoxia (HH) can increase mitochondrial electron leakage, activate xanthine oxidase pathways (XO), stimulate sympathetic activity, and promote inflammatory signaling, thereby contributing to ROS generation [15,16]. Accordingly, ski mountaineering competitions represent a unique model in which exercise- and hypoxia-induced mechanisms coexist and may interact synergistically [17,18].

Against this background, the aim of the present study was to address these gaps by conducting field-based assessments on athletes from the Italian Army Sports Center (Centro Sportivo Esercito Italiano) before and after a ski mountaineering competition. Specifically, this study aims to characterize the integrated physiological, inflammatory, hormonal, and psychological responses of military athletes to high-altitude ski mountaineering competition, hypothesizing that such extreme endurance events elicit significant multidimensional stress responses.

## 2. Materials and Methods

### 2.1. Snapshot of the “Trofeo Mezzalama”

**Figure 1:**
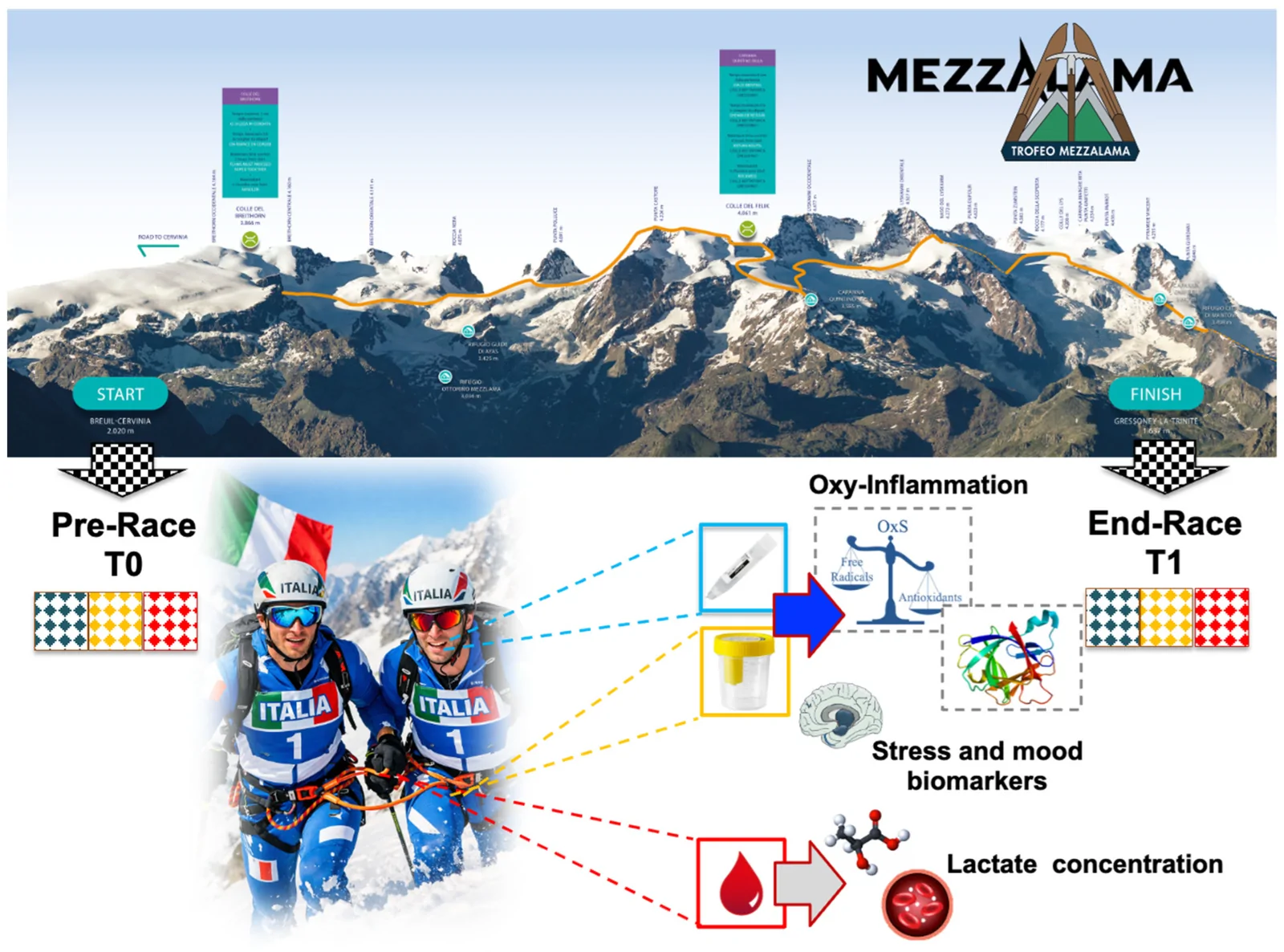
Trofeo Mezzalama elevation profile (Modified by https://www.trofeomezzalama.it, accessed on 14 January 2026). Schematic of the adopted experimental timeline. The right panel shows the biomarkers assessed on the collected biological samples (saliva–square with dark blue diamonds, urine–square with yellow diamonds, and capillary blood–square with red diamonds).

The study was performed during the XXIV Trofeo Mezzalama, held on 26 April 2025. This event is an approximately 45 km ski mountaineering race, starting in Breuil-Cervinia (5:30 a.m.) and finishing in Gressoney-La-Trinité, crossing the Monte Rosa massif in the Aosta Valley and reaching peaks such as Castore (4226 m) and the “Naso del Lyskamm” (4150 m), with a total elevation gain exceeding 4000 m (Figure 1).

The Trofeo Mezzalama, known as the “marathon of the glaciers”, is a historic ski mountaineering race established in 1933 in honor of Ottorino Mezzalama, a pioneer of the ski mountaineering discipline. The competition preserves the original spirit of alpine exploration as it is conducted without mechanical assistance. After several interruptions, the race was permanently revived in 1997 by the Fondazione Trofeo Mezzalama. A distinctive feature of this event is that athletes compete in roped two-person teams, requiring not only exceptional physical endurance but also technical skill, coordination, and continuous interpersonal synchronization. These characteristics, combined with prolonged exposure to high altitude, extreme environmental conditions, and sustained effort, make the Trofeo Mezzalama one of the most demanding ski mountaineering competitions worldwide. From a scientific perspective, the choice of Trofeo Mezzalama as a case study provides a unique and ecologically valid model to investigate integrated physiological and psychological responses under real-world extreme conditions. Compared with laboratory-based protocols or shorter competitions, this event simultaneously exposes athletes to hypoxia, cold stress, prolonged energy expenditure, and complex team dynamics, thereby allowing the assessment of multidimensional stress responses. This makes it particularly relevant for studying military athletes, whose performance often depends on the ability to cope with combined physical and psychological stressors over extended periods.

### 2.2. Participants

A total of 640 athletes, divided into 320 teams, participated in the XXIV Trofeo Mezzalama, won by the Italian team (Italian Army—Esercito Italiano) in a time of 4 h 29 min 19 s. All measurements were performed at rest before (T0) and immediately after (T1) race completion (Figure 1). Nine experienced Italian Army ski mountaineers—four females and five males—collectively designated as T0_all—were enrolled. After receiving information about the experimental procedures, all participants provided written informed consent. The procedure was conducted in accordance with the Declaration of Helsinki, and approval was obtained from the institutional Ethics Committee of the Provinces of Chieti and Pescara (document No. 18; 29 July 2021), Italy.

For each participant, a comprehensive assessment of the health status (i.e., diabetes, allergies, hypertension, hypercholesterolemia, smoking habits), skiing experience, years of training, and supplement use was conducted. All athletes were healthy and non-smokers.

### 2.3. Physiological and Anthropometric Measures

The anthropometric parameters were assessed using bipolar bioelectrical impedance analysis (TBF-300A Body Composition Analyzer; Tanita Corporation, Arlington Heights, IL, USA). Blood pressure (BP) was measured by a standard cuff sphygmomanometer, Peripheral Oxygen Saturation and Heart Rate (SaO_2_ and HR; Oximetry—Ohmeda TuffSat—GE Healthcare, Helsinki, Finland) were also measured (see Table 1). These assessments offered a comprehensive overview of the participants’ baseline characteristics and their acute physiological responses to the endurance event.

### 2.4. Sample Collection

Saliva and urine samples were collected from all participants the day before the competition (at about 12:00 a.m.) and immediately after the end of the race (upon the arrival of each participant). The timing of the baseline sampling was specifically selected to minimize potential confounding effects of the cortisol circadian rhythm.

Baseline samples were therefore collected approximately 24 h before the competition, that is, at approximately the same hour as the expected post-race sampling. In this way, cortisol pre- and post-competition measurements were obtained under similar circadian conditions. Saliva samples were collected using Salivette collection devices (Sarstedt Nümbrecht, Germany). All samples were centrifuged for 5 min at 1000× *g* and aliquoted. Urine samples were collected by voluntary voiding into sterile containers. Samples were maintained at 4 °C in a portable cooler during transportation to the IFC-CNR laboratory in Milan, where they were aliquoted and stored at −80 °C until analysis. Samples were thawed only once, immediately prior to assay. In addition, 20 μL of capillary blood was taken from the fingertip of each recruited athlete.

### 2.5. Biomarkers

The selected biomarkers represent well-established and complementary indicators of the main physiological pathways activated during prolonged exercise and environmental stress.

#### 2.5.1. Metabolic Marker: Lactate

Capillary blood was obtained from the fingertip to determine blood lactate concentration ([La]b) by an enzymatic method (Lactate Scout, InfraTec Diagnostic Solutions S.r.l., Ferrara, Italy) [19,20].

#### 2.5.2. Salivary Determinations

(i) Electron paramagnetic resonance (EPR, E-Scan, Bruker Co., Billerica, MA, USA) technique, operating at 9.3 GHz, was used to determine the ROS production rate. Saliva samples were stabilized at 37 °C using a Temperature and Gas Controller “Bio III” unit (Noxigen Science Transfer & Diagnostics GmbH, Elzach, Germany), interfaced to the spectrometer.

The CMH spin probe (1-hydroxy-3-methoxycarbonyl-2,2,5,5tetramethyl-pyrrolidine-hydrochloride, Noxygen Science Transfer & Diagnostics, Elzach, Germany) (1 mM) was used to trap the unpaired electrons, thereby generating the EPR signal, and the stable CP• (3-Carboxy-2,2,5,5-tetramethyl-1-pyrrolidinyloxy) compound was used as a reference to convert the integrated peak areas of the acquired spectra into absolute ROS concentration (μmol·min^−1^). The method has previously been shown to exhibit high reproducibility [21,22].

(ii) Total Antioxidant Capacity (TAC): the Trolox-equivalent antioxidant capacity assay, a widely used commercial method (Cayman Chemical, Ann Arbor, Michigan, 48108, USA, Item No. 709001), was used to measure total antioxidant capacity. The procedure was carried out in accordance with the manufacturer’s instructions [23].

(iii) Inflammation biomarkers: salivary TNF-α and sICAM-1/CD54 concentrations were determined by ELISA immunoassays (Fine Test, Wuhan, China, Cat No.: EH0302, EH0161), according to the manufacturer’s instructions [24,25]. The assays employ a double-antibody sandwich immunoassay technique. Absorbance was measured at 450 nm, and analyte concentrations were derived from the corresponding standard curves. The coefficients of variation (CV) reported by the manufacturer were inter-assay CV: 5.85% and 4.49%; intra-assay CV: 5.99% and 3.86%, for TNF-α and sICAM-1/CD54, respectively.

(iv) Neurobiological stress markers: salivary cortisol concentrations were determined using enzyme-linked immunosorbent assay (ELISA) kits (cat. No.: EH0641 FineTest, Wuhan, China), following the manufacturer’s instructions [23,26,27]. The inter-assay coefficient of variation was reported by the manufacturer: <10%.

#### 2.5.3. Urinary Determinations

(i) Lipid peroxidation was assessed in urine by measuring 8-isoprostane concentration (8-iso-PGF2α) (Cayman Chemical, Ann Arbor, Michigan 48108, USA, Item No. 516351). The method has been described in previous studies [26,27]. The samples were read at 412 nm wavelength and the concentration was determined using standard curves. Coefficient of variation (CV): inter-assay CV 11.2%; intra-assay CV 14.6%.

(ii) Inflammation biomarker: urinary IL-6 concentration was quantified using ELISA immunoassays (Fine Test, Wuhan, China, Cat No.: EH0201), according to the manufacturer’s instructions [28]. The assay is based on a double-antibody sandwich technique. Sample concentrations were quantified by measuring absorbance at 450 nm. Coefficient of variation (CV): inter-assay CV 1.76%, and intra-assay CV 1.74%.

(iii) Metabolic and Immune Activation indicators: urinary creatinine and neopterin concentrations were quantified using isocratic high-pressure liquid chromatography (HPLC). Linear calibration curves were used for creatinine (1.25–10 mmol/L), neopterin (0.125–1 μmol/L), and uric acid (3.75–60 mmol/L). Inter-assay and intra-assay coefficients of variation were below 5%. The methodology has been described previously [28].

(iv) Neurobiological marker of mood-regulatory responses: urinary serotonin concentration was measured using an enzyme-linked immunosorbent assay kit (cat. No.: BA E-8900 LDN GmbH, Bayern, Germany), following the manufacturer’s instructions [29]. Coefficient of variation (CV): inter-assay CV 11.7%, and intra-assay CV 9.2%.

Urinary 8-isoprostane and neopterin concentrations were normalized to urinary creatinine to account for variations in urine concentration, as previously described [27], since a 24 h urine collection was not feasible.

All samples were analyzed in duplicate, and the spectrophotometric measurements were performed using a microplate reader (Infinite M200, Tecan Group Ltd., Männedorf, Switzerland).

### 2.6. Psychometric, Physical and Subjective Scale

Before and after the competition, the psychometric and physical scales were evaluated, providing a comprehensive overview of both cognitive and physical functions. Each scale was selected on the basis of its reliability and validity in measuring specific attributes. Furthermore, the selected psychometric and perceptual scales are among the most validated and frequently adopted instruments in exercise and sport physiology-psychology.

The following scales were pre competition administered to assess psychological state, and stress levels: the State-Trait Anxiety Inventory (STAI-Y State) to evaluate situational anxiety [30,31], the Perceived Stress Scale—10 items (PSS-10) to measure perceived stress over the past month [32,33]; the Profile of Mood States (POMS) to evaluate the dominant emotional states of elite athletes and non-athletes individuals. In its short form, this instrument was used to assess specific psychological dimensions, including tension–anxiety, depression–dejection, anger–hostility, vigor–activity, fatigue–inertia, and confusion–bewilderment, with each dimension rated on a five-point scale ranging from 0 to 4 [4,34]. High vigor scores indicate more positive mood states or emotions, whereas low scores reflect a negative mood or emotion.

The Total Quality of Recovery (TQR) scale, proposed by Kenttä and Hassmén [35,36], was also assessed to evaluate athletes’ perceived recovery status.

Post-competition, the perceived exertion based on physical sensations was determined as follows: muscle fatigue using the Borg Rate of Perceived Exertion scale (RPE) [37], and the Rating of Fatigue scale (ROF) to monitor perceived fatigue intensity [38].

The Verbal Rating Scale for Stress (VRS) to quantify subjective stress related to the upcoming event was also administered [39]. Finally, pre- and post-competition, a 0–100 mm visual analog scale (VAS) was employed to evaluate subjective mood, overall well-being (happy/unhappy, rested/tired), general sensations (hot/cold, calm/agitated), and the presence or absence of head pain. When pain was reported, its location was also recorded [4,40].

Altogether, these physiological, biochemical, and psychometric measures provide an integrated assessment of oxidative stress, inflammation, neuroendocrine activation, immune function, fatigue, recovery, and psychological responses during prolonged endurance exercise.

### 2.7. Statistical Analysis

Statistical analysis and graphical representations were conducted using the GraphPad Prism software for Mac (GraphPad Software Inc., California, USA, version 11.0.0, https://www.graphpad.com, access 14 January 2026) and the statistical package IBM SPSS Statistics (IBM SPSS Statistics v. 22, Armonk, NY, USA) software. Data normality was evaluated using the Shapiro–Wilk test, followed by paired-samples *t*-tests. Percentage changes, defined as (post-pre/pre competition)*100), were used for the biomarkers analysis. Confidence intervals (CI 95%) for the estimated values was calculated and d-Cohen was used for calculating the size effect. A regression analysis was performed to assess the association between completion time (minutes) and ROS production rate. Both linear and second-order polynomial models were fitted; a non-linear relationship was established. The data are expressed as mean ± standard deviation (SD). Statistical significance was set at *p*-value < 0.05. The figures show Bean plots representing dataset distribution, shape, spread, and variability. The bean width reflects data density, allowing visualization of distribution patterns and differences between groups.

## 3. Results

Of the nine initially enrolled ski mountaineers (T0_all), only seven completed the competition, reaching Gressoney La-Trinitè, and were re-evaluated at T1 (see Table 1). None of the subjects were taking antioxidant supplements and/or had assumed any medication in the 72 h preceding the race.

### 3.1. Metabolic Marker: Lactate

Compared to T0, a significant increase in lactate concentration was measured at T1: 8.98 ± 2.27 vs. 1.50 ± 0.63 mM, reflecting the exercise-induced acute metabolic response (see Table 1).

### 3.2. Oxi-Inflammation Biomarkers

**Figure 2:**
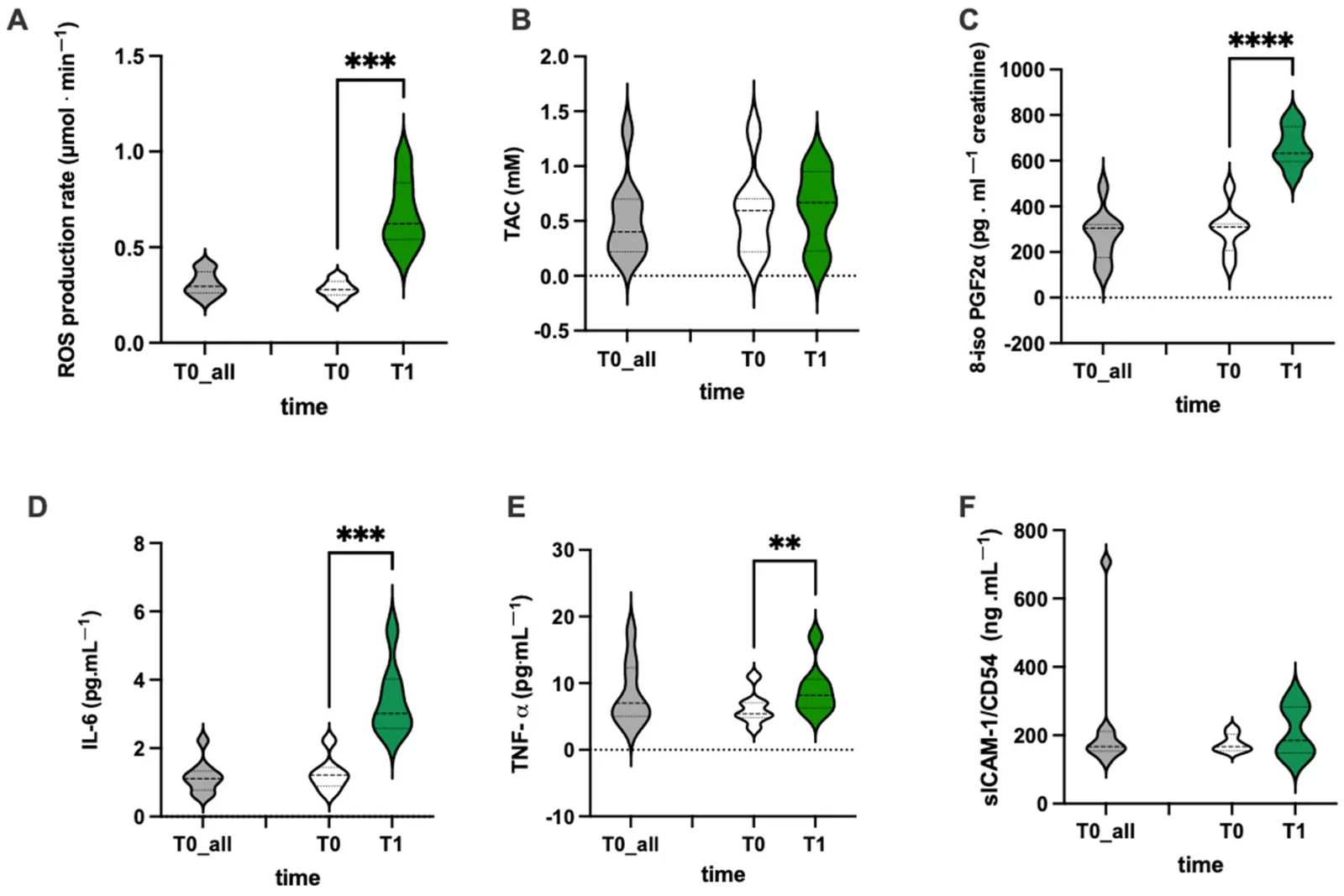
Bean plots of oxy-inflammation parameters from all the recruited subjects (T0_all: *n* = 9) and only the finishers at T0 and T1 (*n* = 7): (**A**) ROS production rate: (μmol·min^−1^); (**B**) antioxidant capacity (TAC, mM); (**C**) 8-isoprostane (8-iso-PGF2α, pg·mg^−1^creatinine); (**D**) Interleukin 6 (IL 6, pg·mL^−1^); (**E**) TNF-α (pg·mL^−1^); and (**F**) s-ICAM (ng·mL^−1^). Significance levels: ** *p* < 0.01, *** *p* < 0.001, and **** *p* < 0.0001.

Oxidative stress biomarkers were found to be significantly increased at T1. ROS production rate significantly increased at T1 vs. T0: 0.67 ± 0.17 vs. 0.28 ± 0.04 μmol·min^−1^; +137%; d-Cohen: 3.158; CI 95%: 1.588–4.728; *p* < 0.001 (Figure 2A). By contrast, TAC did not show a significant increase at T1 vs. T0: 0.61 ± 0.35 vs. 0.58 ± 0.39 mM; +4%; *p* > 0.05 (Figure 2B).

Lipids peroxidation (8-iso-PGF2α), significantly increased at T1 vs. T0: 661.00 ± 89.93 vs. 298.70 ± 105.70 pg·mg^−1^creatinine; +121%; d-Cohen: 3.692; CI 95%: 1.969–5.415; *p* < 0.0001; (Figure 2C); associated with changes in systemic inflammation, IL-6: T1 vs. T0: 3.41 ± 1.05 vs. 1.24 ± 0.49; +175%; d-Cohen: 2.649; CI 95%: 1.213–4.084; *p* < 0.001 (Figure 2D); TNF-α: T1 vs. T0 9.09 ± 3.92 vs. 6.26 ± 2.45; +45%; d-Cohen: 0.866; CI 95%: 0.23–1.96; *p* < 0.01 (Figure 2E); while, no significant changes in sICAM-1/CD54: T1 vs. T0: 212.20 ± 75.39 vs. 177.30 ± 25.68; +20%; *p* > 0.05 (Figure 2F).

### 3.3. Stress and Mood Regulation Molecules

**Figure 3:**
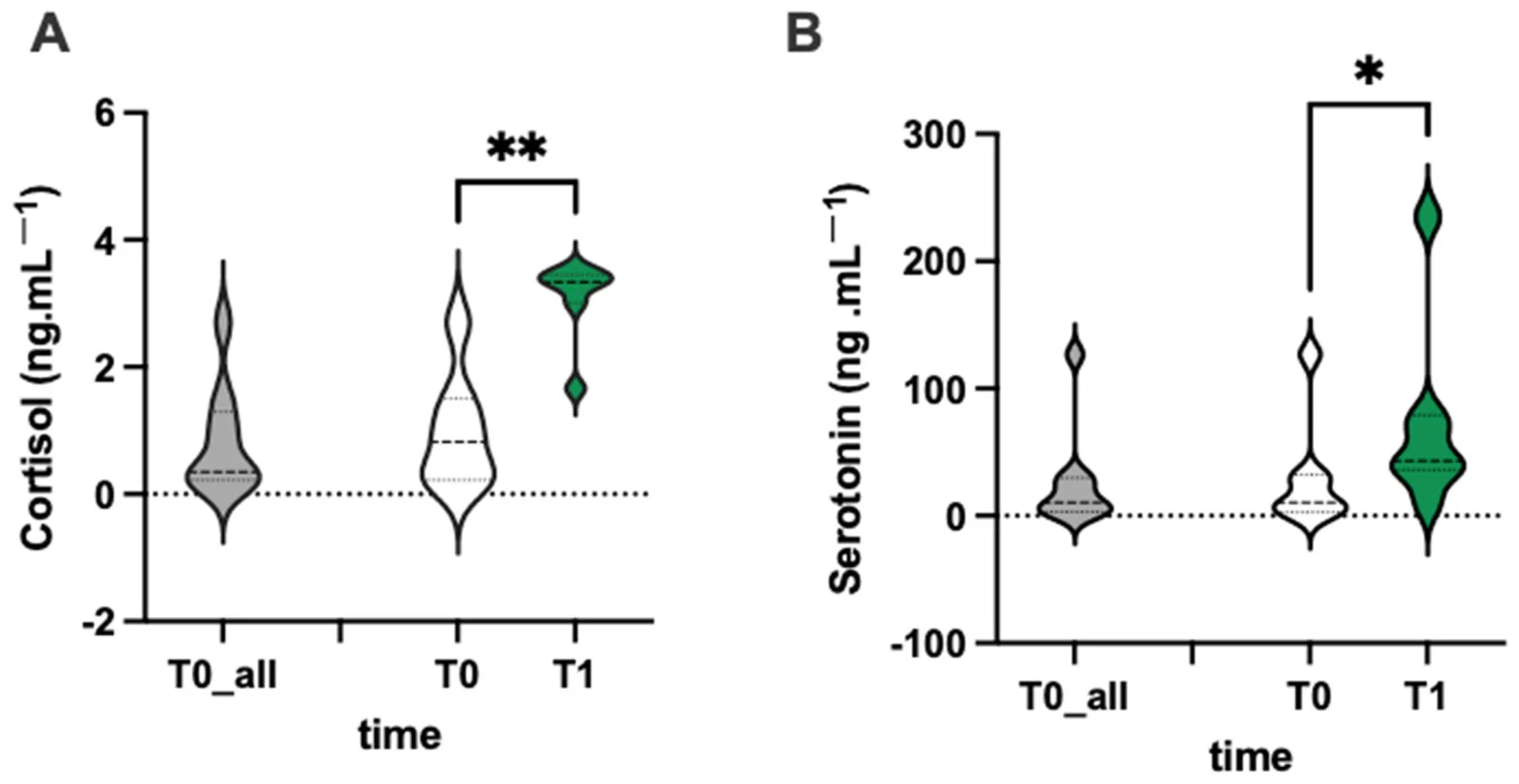
Bean plots of psychophysiological stress biomarker concentrations from all the recruited subjects (T0_all: *n* = 9) and only the finishers at T0 and T1 (*n* = 7): (**A**) cortisol and (**B**) serotonin (ng·mL^−1^). Significance levels: * *p* < 0.05, ** *p* < 0.01.

A significant increase in cortisol hormone at T1 vs. T0 was found: 3.09 ± 0.65 vs. 0.94 ± 0.89 ng·mL^−1^; +214%; d-Cohen: 2.759; CI 95%: 1.295–4.222; *p* < 0.01 (Figure 3A); and serotonin levels 72.65 ± 74.97 vs. 30.14 ± 44.17 ng·mL^−1^; +141%; d-Cohen: 0.691; CI 95%: 0.388–1.769; *p* < 0.05 (Figure 3B); suggesting an exercise-induced coordinated changes in neuroendocrine responses and serotonergic mechanisms involved in mood regulation.

### 3.4. Renal Status Biomarkers and Immune System Activation

**Figure 4:**
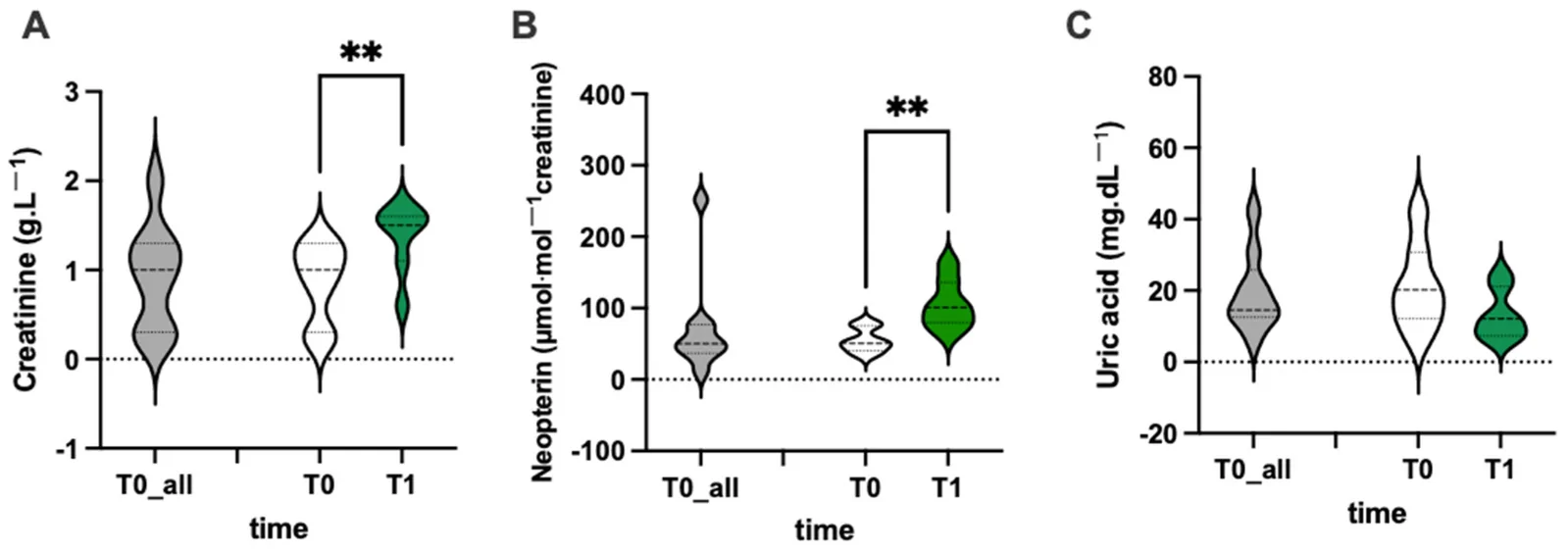
Renal status biomarkers: Bean plots from all the recruited subjects (T0_all: *n* = 9) and only the finishers at T0 and T1 (*n* = 7). (**A**) creatinine (g·L^−1^); (**B**) neopterin (μmol.mol^−1^creatinine); (**C**) uric acid concentrations detected in urine. Significance level: ** *p* < 0.01.

Renal status and immune system activation markers showed significant increases in creatinine and neopterin levels, respectively, at T1 vs. T0. Creatinine: 1.37 ± 0.39 vs. 0.85 ± 0.45 g·L^−1^; +60%; d-Cohen: 1.235; CI 95%: 0.092–2.378; *p* < 0.01 (Figure 4A), and neopterin/creatinine: 104.90 ± 34.45 vs. 54.20 ± 17.07 μmol·mol^−1^creatinine; +94%; d-Cohen: 1.865; CI 95%: 0.61–3.12; *p* < 0.01 (Figure 4B), suggesting an acute exercise-induced response affecting renal function and immune activation. No significant differences in uric acid were found at T1 vs. T0: 13.17 ± 7.07 vs. 21.01 ± 12.12 g·L^−1^; *p* > 0.05 (Figure 4C).

### 3.5. Psychometric, Physical and Subjective Scale

The psychometric, physical, and subjective scales administered only at T0 showed the following scores: STAY-Y state: 46.87 ± 2.32: moderate-to-high state anxiety level; PSS-10: 19.75 ± 5.38: moderate level of perceived stress; POMS: 18.0 ± 9.86, low-to-moderate mood disturbance; TQR: 13.75 ± 0.88: all subjects had achieved a reasonable recovery state.

**Figure 5:**
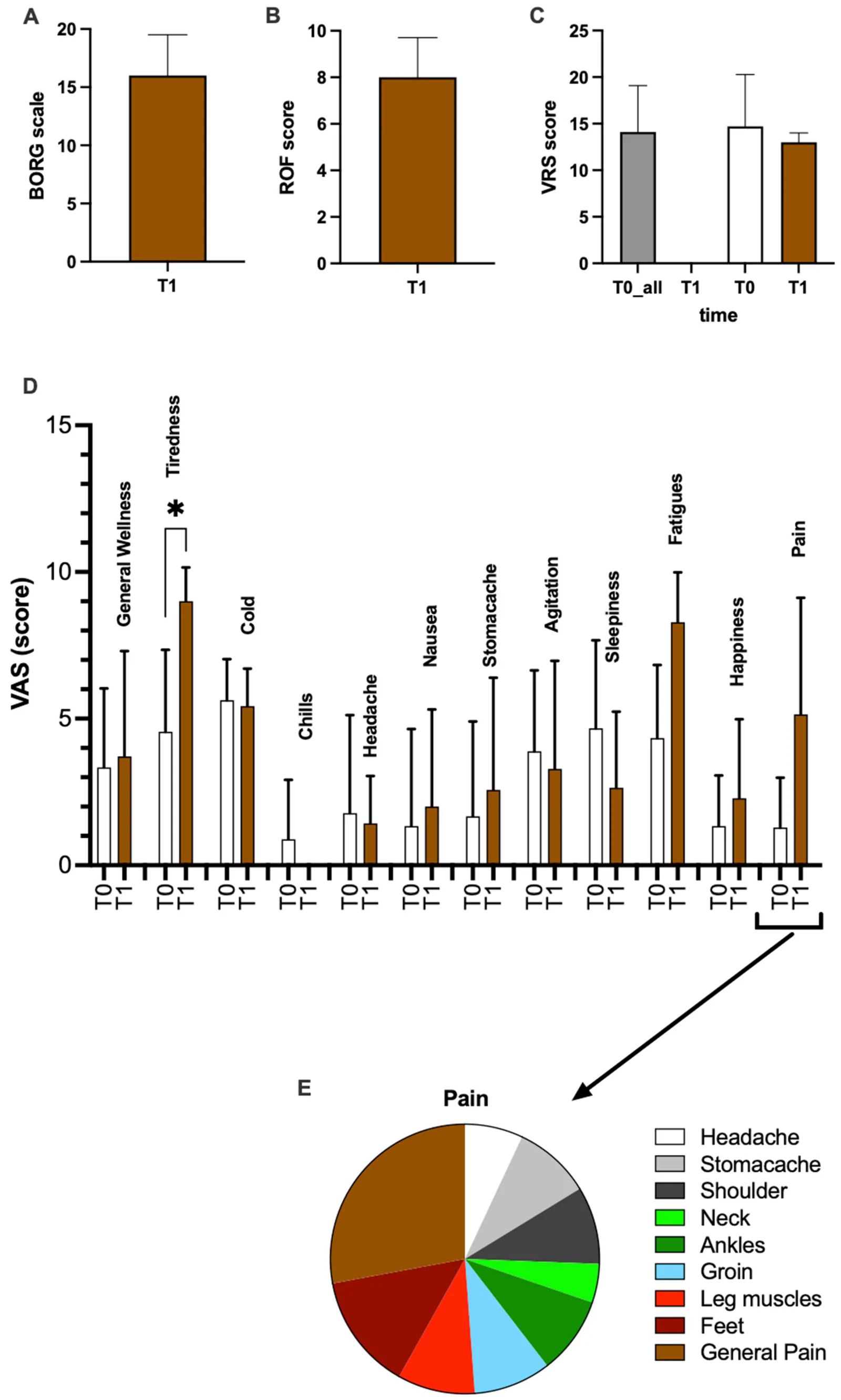
Psychometric, physical, and subjective scales. Histogram plots of: (**A**) Borg, (**B**) ROF, (**C**) VRS, and (**D**) VAS scores for general wellness, tiredness, cold, chills, headache, nausea, stomachache, agitation, sleepiness, fatigue, happiness, and pain at T0 and T1. Statistically significant difference comparisons are displayed: * *p* < 0.05. In (**E**), the expansion chart depicts the relative distribution of pain areas at T1 vs. T0, providing a visualization of the part-to-whole relationships among the affected anatomical regions.

Figure 5 reports changes in Borg scores with a mean of 16.16 ± 3.51 scores (Figure 5A); change in ROF score with a mean of 8.08 ± 1.70 (Figure 5B); no significant differences in VRS at T1: 14.71 ± 5.55 vs. 6.33 ± 0.69 (Figure 5C). Finally, the VAS item score pre vs. post is reported in Figure 5D. T1 vs. T0 percentage changes: rested/tiredness +98%; fatigue +112%; happiness +36%; pain +320%. Pain intensity was significantly higher at T1 than at T0, indicating a marked increase immediately following exercise. In Figure 5E, the expansion chart shows the percentages of body segments affected by pain. Overall, pain accounts for the highest percentage (86%); it can be primarily described as general pain and widespread physical discomfort. Foot pain is also high (83%), likely associated with the use of ski boots. Only one case (14%) of pain in the neck/cervical region is reported.

### 3.6. Competition Time and ROS Relationship

**Figure 6:**
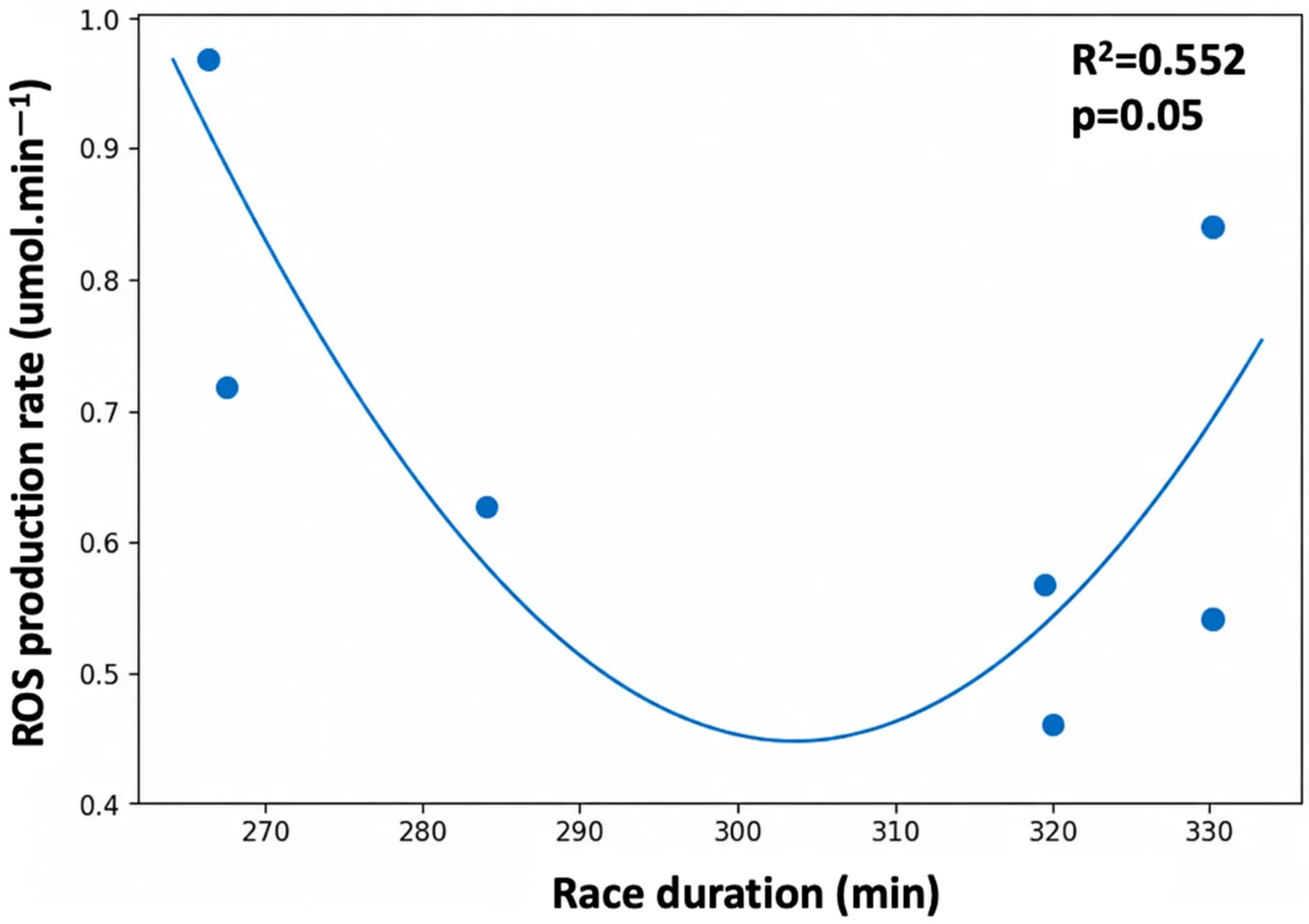
Relationship between ROS and race duration time: the blue line shows the quadratic regression fit curve. The correlation coefficient (R^2^) is reported. All data points represent individual observations.

The relationship between ROS production rate and T1 (race duration—minutes) is reported in Figure 6. The fitted second-order polynomial model (R^2^ = 0.552, *p* = 0.05) suggests a potential non-linear pattern, characterized by lower ROS production rates at intermediate race-time values. However, given the limited number of observations, this pattern should be cautiously interpreted and considered exploratory.

## 4. Discussion

At the end of the Mezzalama competition, all subjects exhibited a significant increase in oxidative stress associated with a mild inflammatory response. The observed increase in ROS production likely reflects the combined influence of prolonged endurance exercise and high-altitude exposure. Exercise is known to increase mitochondrial oxygen flux and activate enzymatic ROS-producing systems, including muscle NADPH (nicotinamide adenine dinucleotide phosphate), whereas HH may further enhance oxidative stress through mitochondrial electron transport chain perturbation, sympathetic activation, and redox-sensitive inflammatory signaling pathways [19,20,41,42]. As a matter of fact, both stressors simultaneously occurred throughout the competition; their individual contributions cannot be distinguished in the present study. On the other hand, after prolonged exercise, to counteract ROS increase, the body is known to increase its systemic antioxidant capacity, but it is not always sufficient to buffer the free-radical effects on lipids and cellular compartments [43,44].

This observation is consistent with evidence showing that repeated muscle activation can promote adaptive changes in skeletal muscle oxidative capacity, suggesting that moderate ROS production may contribute to beneficial physiological adaptations rather than representing exclusively a deleterious process [45,46].

Our data show a significant and important increase in membrane lipid damage associated with systemic post-race inflammation. IL-6 can increase in ultramarathons [28,47], and the magnitude of the increase is related to the duration of the effort and the distance traveled. The marked increase in IL-6 is consistent with the well-established role of contracting skeletal muscle as a major source of exercise-induced IL-6 release. Although hypoxia may contribute to inflammatory signaling, the magnitude of the response observed here is likely driven primarily by the prolonged muscular workload [48]. TNF-α generally increases less in well-trained athletes, even if it can be found significantly elevated in conditions of extreme fatigue, and/or dehydration, and/or metabolic stress [49,50].

Endothelial activation induced by hypoxic exposure may also contribute to the increase in adhesion molecules such as sICAM-1, which has been previously reported during altitude exposure and reflects vascular inflammatory activation. Therefore, while the increase in IL-6 observed in the present study is likely primarily driven by the prolonged muscular workload, the elevation of sICAM-1 may also reflect the contribution of high-altitude hypoxic stress [51].

During endurance exercise, emerges a characteristic neuroendocrine pathway profile activated during physical and psychological stress to maintain physiological homeostasis. After prolonged exercise, cortisol levels increase immediately. Indeed, experimental studies have shown that endurance exercise stimulates the hypothalamic–pituitary–adrenal axis (HPA), leading to a measurable rise in circulating cortisol concentrations, especially when exercise intensity exceeds a certain metabolic threshold [52]. Cortisol elevation likely reflects activation of the HPA by both prolonged exercise and environmental hypoxic stress [53,54]. Previous studies have shown that acute altitude exposure can independently stimulate cortisol secretion owing to sympathetic activation and increased physiological strain. Therefore, the hormonal response observed after the Mezzalama competition is likely multifactorial [55,56,57]. Evidence from marathon studies confirms this response. For example, salivary cortisol measurements taken during marathon races show that cortisol levels progressively increase throughout the race and often peak shortly after completion, reflecting sustained activation of adrenal secretion during prolonged exertion [58]. Moreover, cortisol responses appear to be associated with perceived fatigue and metabolic stress. During prolonged exercise, cortisol helps maintaining blood glucose availability by stimulating gluconeogenesis and mobilizing energy substrates such as amino acids and free fatty acids. In endurance athletes, acute exercise can elevate cortisol production to levels comparable to those observed in some endocrine stress conditions, although with typically transient increases [59]. Furthermore, prolonged physical stress provides another variable to be particularly studied in military contexts. In fact, during extended training exercises, sustained physiological stress and elevated cortisol responses have been associated with reductions in cognitive performance and operational efficiency in military personnel [60].

It is well known that after competitions or intense exercise, changes can occur in brain neurochemistry, involving several neurotransmitters, including serotonin (5-HT). Although serotonin is not considered a direct biomarker of the physiological stress response, it plays an important role as a neurobiological mediator involved in mood regulation, emotional state, and adaptation to physical exercise [61]. Prolonged or high-intensity physical activity alters the balance of central neurotransmitters through metabolic and neuroendocrine mechanisms that affect the central nervous system (CNS) [62]. One widely accepted explanation is the central fatigue hypothesis, proposed by Eric A. Newsholme (2006) [63], suggesting that prolonged exercise increases the availability of tryptophan, the precursor of serotonin, in the brain. During endurance exercise, increased lipolysis raises circulating free fatty acids, which, displacing tryptophan from albumin, increases the free tryptophan plasma concentration [64,65], thereby facilitating its transport across the blood–brain barrier and leading to an increase in serotonin synthesis in the brain [66,67]. Although increased urinary serotonin may reflect altered serotonergic metabolism following prolonged exercise, urinary measurements cannot be directly interpreted as indicators of CNS serotonin activity [64]. Therefore, any association with central fatigue mechanisms should be considered speculative.

A significant increase in creatinine and neopterin levels was also found, whereas uric acid concentrations remained relatively constant. The rise in creatinine is commonly observed after prolonged or intense exercise: it is generally associated with increased muscle metabolism, transient muscle breakdown, and reduced renal perfusion [68]. A similar increase is frequently reported in endurance events, particularly in marathon and ultramarathon runners [19,20,28,69,70]. Similarly, neopterin levels showed a significant increase, suggesting activation of the cellular immune response; in fact, neopterin is produced by macrophages following stimulation by interferon-γ and is widely used as a marker of immune activation and oxidative stress, both of which may occur after prolonged physical exertion [71].

Immediately after the competition (T1), athletes reported subjective ratings of perceived exertion on the Borg scale with a mean of 15 (scores), along with a fatigue rating (ROF) of 8 (scores), reflecting a high level of overall exertion and tiredness. They also described generalized fatigue, accompanied by localized discomfort predominantly in the lower limbs, including muscle soreness in legs and feet. These symptoms are likely related to muscle fatigue, environmental factors such as heat or cold, and prolonged mechanical stress on the feet and lower extremities at the end of the competition. Muscle soreness after intense exercise, particularly in the lower limbs following marathon and ultramarathon efforts, is consistent with the clinical description of delayed onset muscle soreness (DOMS), reflecting mechanical strain and microtrauma to muscle fibers [19,72,73].

The observed association between race competition time and ROS production (R^2^ = 0.55, *p* = 0.05) suggests a potential nonlinear pattern in the oxidative stress response rather than a simple linear increase with the performance time. Although this pattern is biologically plausible and may be consistent with the concept of redox hormesis, it should be cautiously interpreted, given the small sample size, and considered exploratory and hypothesis-generating. Further studies with larger cohorts are needed to determine whether differences in exercise duration, intensity, and cumulative physiological stress could contribute to such a biphasic response. Powers et al. (2020) described ROS responses to exercise as inherently biphasic, where low-to-moderate ROS levels act as signaling molecules promoting adaptation, while excessive ROS can impair function and increase oxidative damage [74].

An alternative explanation is that athletes with longer race times experienced a greater cumulative exposure to hypoxia and environmental stressors, which may have contributed to the elevated ROS levels observed in the slower finishers. Consequently, the observed non-linear pattern may reflect the interaction between exercise intensity and the duration of altitude exposure rather than exercise intensity alone [14]. From a performance perspective, the higher ROS values observed in the fastest athletes may reflect the substantial metabolic demand, greater oxygen flux, catecholamine release, and muscle fiber recruitment associated with elite race intensity [75]. Finally, high-intensity exercise is well known to acutely increase mitochondrial and non-mitochondrial ROS generation [76].

### Limitation

Given the limited sample size (*n* = 7), caution is warranted in interpreting the final conclusions of the manuscript. We included all Italian Army athletes who participated in the competition; however, despite the model reaching statistical significance, the estimates remain highly sensitive to outliers and should therefore be considered exploratory in nature. Future studies with larger cohorts and additional covariates (i.e., VO_2_ max, training load, hydration, nutrition, effects of environmental temperature on bio-physiological performance) are needed to confirm whether post-race ROS levels truly follow a non-linear relationship with performance in extreme endurance skiing competitions.

Potentially, the findings of this study have important practical implications for both military personnel and endurance athletes exposed to prolonged strenuous exercise under environmental stress. The post-competition changes observed in oxidative stress, inflammatory mediators, and psychometric responses indicate that integrated bio-physio-psychological monitoring could be helping to identify individuals exposed to risk of excessive fatigue, impaired recovery, or maladaptation. In agreement with our hypothesis, recent evidence suggests that circulating biomarkers, including cortisol, neuropeptide-Y (NPY), insulin-like growth factor-1 (IGF-1), inflammatory mediators, and oxidative stress markers, can provide objective indicators of physiological resilience and operational readiness in military personnel, supporting individualized training and recovery strategies [77,78]. Furthermore, metabolomic studies conducted in soldiers undergoing Special Forces selection have shown that individuals with lower oxidative stress profiles and better nutritional status exhibit superior physical performance and a greater likelihood of successful race completion, highlighting the importance of monitoring redox balance before and during demanding training periods [79]. Finally, in military settings, integrating non-invasive biomarkers with psychometric assessments may assist commanders and medical personnel in optimizing workload distribution and operational readiness. Similarly, for endurance athletes, combining biochemical and psychological assessments may facilitate individualized training prescriptions, early detection of excessive physiological strain, and targeted nutritional or recovery interventions aimed at minimizing oxidative damage and promoting optimal adaptation [80]. Recently, systematic reviews also supported the use of salivary biomarkers as practical point-of-care tools for monitoring readiness in athletes operating under sleep deprivation, dehydration, and prolonged physical stress [81,82].

## 5. Conclusions

Ski mountaineering competitions place an extremely high physiological burden on athletes, eliciting complex oxidative, inflammatory, metabolic, hormonal, neuroendocrine, and psychophysiological responses.

The findings of the present study showed that high-altitude ski mountaineering competition imposes significant oxidative, inflammatory, and psychological stress on military athletes, underscoring the need for targeted recovery and adaptation strategies. The observed pattern suggested a potential nonlinear association between post-competition ROS production and performance time, possibly reflecting the complex interplay between exercise intensity, cumulative hypoxic exposure, and redox adaptive mechanisms. However, given the small sample size, this finding should be considered exploratory and hypothesis-generating rather than evidence of a definitive non-linear pattern of response, warranting confirmation in larger cohorts. Nevertheless, in our opinion, studies conducted in extreme environments often face unavoidable recruitment limitations, and even small cohorts may provide meaningful physiological information. Furthermore, the longitudinal within-subject design, with repeated measurements collected under controlled conditions, partially mitigated the impact of inter-individual variability, providing valuable insights into dynamic physiological responses to extreme endurance stress.

Future studies will include larger, more diverse cohorts and consider additional variables such as VO_2_ max, training load, and environmental conditions.

## Figures and Tables

**Table 1:** Mean (±SD) of the parameters collected from the athletes taken altogether at Pre (T0_all *n* = 9); and/or pre-post competition (T0 and T1 *n* = 7). BMI: Body Mass Index; SaO_2_: arterial Oxygen Saturation. HR: Heart Rate. Significant differences when compared to pre-race: * *p* < 0.05.

	T0_all (*n* = 9) Mean ± SD	T0 (*n* = 7) Mean ± SD	T1 (*n* = 7) Mean ± SD
**Age (years)**	32.37 ± 8.90	30.14 ± 6.12	-
**Body weight (kg)**	59.37 ± 9.58	62.57 ± 9.16	-
**Height (cm)**	172.12 ± 8.33	174.28 ± 8.34	-
**BMI (kg·m^−2^)**	19.85 ± 1.53	20.48 ± 1.37	-
**Systolic Blood Pressure (mmHg)**	124.62 ± 11.03	124.30 ± 11.60	189.90 ± 41.03 *
**Diastolic Blood Pressure (mmHg)**	77.50 ± 8.70	77.57 ± 9.36	91.00 ± 7.32 *
**SaO_2_ (%)**	96.87 ± 0.70	96.86 ± 3.04	94.0 ± 1.12 *
**HR (bpm)**	65.75 ± 13.64	68.00 ± 15.38	132.5 ± 22.61 *
**Lactate [La]b(mM)**	1.80 ± 0.87	1.50 ± 0.63	8.98 ± 2.27 *

## Data Availability

All raw data that were presented or mentioned in the manuscript are available from the corresponding author on request.

## Abbreviations

The following abbreviations are used in this manuscript:

CMH	1-hydroxy-3-methoxy-carbonyl-2,2,5,5-tetramethylpyrrolidine
CNS	Central Nervous System
CP	3-carboxy-2,2,5,5-tetramethyl-1-pyrrolidi-nyloxy
DOMS	Delayed Onset Muscle Soreness
EPR	Electron Paramagnetic Resonance
HH	Hypobaric Hypoxia
HPA	Hypothalamic–Pituitary–Adrenal Axis
HPLC	High-Performance Liquid Chromatography
IL- 6	Interleukin-6
NADPH	Nicotinamide Adenine Dinucleotide Phosphate
OxS	Oxidative Stress
POMS	Profile of Mood State
PSS	Perceived Stress Scale
ROF	Rating of Fatigue
RPE	Rate of Perceived Exertion
ROS	Reactive Oxygen Species
sICAM-1/CD54	Soluble Intercellular Adhesion Molecule 1/CD54
TAC	Total Antioxidant Capacity
TNF-α	Tumor Necrosis Factor-alpha
VAS	Visual Analog Scale
8-iso-PGF2α	8-isoprostane-PGF2 alpha
XO	Xanthine Oxidase
